# Association of geriatric nutritional risk index with metabolic dysfunction-associated steatotic liver disease and subtypes in Chinese elderly: identification of an overnutrition risk threshold and implications for extended risk stratification

**DOI:** 10.3389/fnut.2026.1743679

**Published:** 2026-02-13

**Authors:** Meiyan Guo, Lifang Mao, Qiuyun Kang, Haixin Lyu, Haiyan Chen, Yangyang Qin, Zegeng Zhan, Tianran Shen

**Affiliations:** Department of Nutrition and Food Hygiene, School of Public Health, Guangdong Pharmaceutical University, Guangzhou, Guangdong, China

**Keywords:** geriatric nutritional risk index, MASLD subtypes, mediation analyses, metabolic dysfunction-associated steatotic liver disease, nutritional status, risk prediction

## Abstract

**Background:**

Current evidence on the geriatric nutritional risk index (GNRI) and metabolic dysfunction-associated steatotic liver disease (MASLD) in the elderly is inconsistent, with limited data from Chinese studies. Notably, few research has explored the association between GNRI and MASLD subtypes. Therefore, this study aimed to investigate the association between GNRI and the risk of MASLD and subtypes in a Chinese elderly population.

**Methods:**

This cross-sectional study recruited 7,628 Chinese adults aged≥60 years from Zhongshan during 2020-2021. Binary and multinomial logistic regression were used to analyze the associations between GNRI and MASLD prevalence and subtypes, respectively. Restricted cubic splines (RCS) were employed to explore non-linear relationship. Receiver operating characteristic curves and the area under the curve (AUC) were used to evaluate GNRI's predictive accuracy. Mediation and stratified analyses were conducted to explore underlying mechanisms and subgroup effects.

**Results:**

Among 7,628 participants (40.4% male; MASLD prevalence: 38.8%), 96.6% were classified as “no-risk” (GNRI≥98) according to traditional criteria, highlighting the limitation of current risk stratification. Fully adjusted model demonstrated that each unit increase in GNRI elevated MASLD risk by 12% (OR = 1.12, 95% CI: 1.10–1.13), with quartile analysis revealing a dose-dependent increase (Q1: reference; Q2: OR = 1.56, 95% CI: 1.31–1.84; Q3: OR = 1.93, 95% CI: 1.64–2.28; Q4: OR = 2.70, 95% CI: 2.28–3.20; *P*_−trend_ < 0.001). RCS identified a nonlinear inflection at GNRI≥107.59 (*P*_−overall_ < 0.001; *P*_−nonlinear_ = 0.008), where the risk of MASLD escalated substantially. GNRI showed moderate predictive accuracy (AUC = 0.802, 95% CI: 0.792–0.812), while mediation analysis indicated BMI accounted for the largest proportion of the total effect of GNRI on MASLD (21.1%, 95% CI: 6.4%-29.2%). Using the non-MASLD population as a reference, as GNRI levels increased, the risks of MASLD subtypes increased significantly, following a gradient: overweight/obesity subtype>diabetes subtype>lean metabolic disorder subtype (all *P*<0.05).

**Conclusion:**

Elevated GNRI significantly increases MASLD and subtype risks in Chinese elderly, with GNRI ≥107.59 identified as a critical threshold for escalating the risk of MASLD. These findings support extending GNRI's risk stratification to overnutrition monitoring, enabling prioritized screening for metabolic hazards. Future research should validate this threshold's clinical utility, establish evidence-based upper limits for overnutrition risk, and explore GNRI's role in MASLD pathogenesis.

## 1 Introduction

Metabolic dysfunction-associated steatotic liver disease (MASLD), formerly known as non-alcoholic fatty liver disease (NAFLD), is a systemic metabolic disorder characterized by excessive hepatic fat accumulation, insulin resistance, and systemic inflammation (1). It causes a series of hepatic and extrahepatic complications, including but not limited to risks for cirrhosis, hepatocellular carcinoma, cardiovascular disease, type 2 diabetes mellitus, chronic kidney disease, and premature death (2–4). Over the past few decades, the overall global prevalence of MASLD has steadily increased and is currently estimated to be 38% (5, 6). Among Asian countries, China has the highest MASLD prevalence, incidence, and annual MASLD-related mortality (7), highlighting the urgent need for prevention and control strategies in the Chinese population. Notably, MASLD has substantial heterogeneity that can be categorized into distinct subtypes based on metabolic conditions (8, 9). Its prevalence is particularly high among older adults, especially those aged over 60 years (10). However, simple and effective indicators for predicting the risk of MASLD and its subtypes in the elderly Chinese population are still lacking for disease diagnosis, prognosis, and progression monitoring.

Malnutrition, encompassing both undernutrition and over nutrition, exerts a more profound negative influence on the body's systems, thereby impacting the overall health status and quality of life (11). Malnutrition is a highly prevalent condition in older adults, and poses a substantial burden on health, social, and aged-care systems (12). A multitude of studies have furnished us with novel instruments for nutritional screening and assessment, presenting additional choices for evaluating diseases and their prognoses (13). The geriatric nutritional risk index (GNRI), a nutrition-related risk index which is designed to assess the nutritional status of older adults and predict the risk of malnutrition by combining serum albumin levels, actual body weight, and ideal body weight, was initially used to assess the risk of mortality and morbidity associated with malnutrition (14). Numerous researchers have found that GNRI has predictive value for a wide range of diseases and their prognoses, including cirrhosis, hepatocellular carcinoma, pancreatic cancer, hyperlipidemia, and acute myocardial infarction (15–19). To date, only a few studies have explored the association between GNRI and MASLD, and the findings have been inconsistent (20, 21). Moreover, evidence on the relationship between GNRI and MASLD subtypes is lacking, particularly in the Chinese population.

Therefore, this study aimed to investigate the association between GNRI and the risk of MASLD and its subtypes in a Chinese elderly population. Exploring different MASLD subtypes may help better characterize the heterogeneity of the disease and provide a scientific basis for individualized management strategies. The findings of this study are expected to enhance understanding of the role of nutritional status in MASLD among older adults and to inform targeted nutritional interventions for the prevention and management of fatty liver disease in this population.

## 2 Materials and methods

### 2.1 Study population and design

This cross-sectional study, based on the National Basic Public Health Service Program for the elderly and focusing on diseases of glycometabolic and lipid metabolic disorders, recruited 21,025 participants from August 2020 to October 2021 in Minzhong Town and Torch Development Zone, Zhongshan City, Guangdong Province. The recruitment was carried out through local advertisements, invitations, health lectures, or community referrals. All participants had no history of severe infectious diseases, terminal malignancies, recent major surgeries, or trauma and had resided locally for over six months. The questionnaire survey was carried out by qualified, trained investigators. Specifically, it was completed on-site in a one-to-one, face-to-face manner with each question posed individually. The questionnaire included questions related to the name, gender, age, ethnicity, education, marital status, smoking and drinking status, and medical history of the study participants. Physical examination, clinical evaluation, and laboratory testing were performed by expert physicians. The study protocol was approved by the Ethics Committee of Guangdong Pharmaceutical University [Medical Ethics (2019) No.109], and all study participants provided written informed consent. All procedures were conducted by the ethical guidelines of the Declaration of Helsinki. This study followed the Strengthening the Reporting of Observational Studies in Epidemiology (STROBE) reporting guideline for cross-sectional studies.

**Figure 1:**
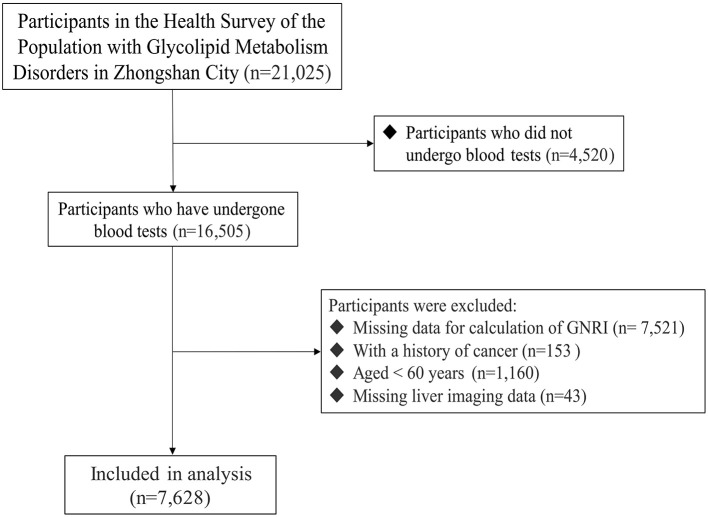
Flowchart illustrating selection of the study population. GNRI, geriatric nutritional risk index.

Participants were excluded under the following conditions: if they did not undergo a blood test (*n* = 4,520), had missing data for calculating the GNRI (*n* = 7,521), had a history of cancer (*n* = 153), were aged less than 60 years (*n* = 1,160), or lacked liver imaging data. Consequently, a total of 7,628 elderly individuals were included in the final analysis (Figure 1).

The sample calculation formula for cross-sectional study is as follows:


$$\begin{array}{l} N=\frac{Z_{\left(1-\alpha \right)/2}^{2}p\left(1-p\right)}{d^{2}} \end{array}$$

*N* denotes the sample size, (Z _(1−α)/2_) is the critical value of the standard normal distribution at the selected confidence level, *p* represents the expected prevalence, and *d* is the allowable error. The current prevalence of MASLD in China is 29.6% (10). Thus, the sample size was calculated with α = 0.05, (Z_(1−α)/2_) = 1.96, *p* = 0.296, *d* = 0.1 × *p* = 0.0296. The final calculated sample size was 914 cases. Taking into account a 20% questionnaire non-response rate, at least 1,143 cases needed to be included in our study. The number of respondents was 21,025 cases.

### 2.2 Diagnosis of MASLD

The diagnostic criteria used in this study are based on those recommended in the Chinese Guidelines for the Diagnosis and Treatment of Fatty Liver Disease (2024 Edition) (22).The diagnostic criteria for MASLD (Chinese standard) were based on hepatic steatosis identified by imaging or biopsy, exclusion of liver injury from other known causes (presence of genotype 3 HCV infection, drug-induced fatty liver, hepatic leguminous nuclear degeneration, and malnutrition leading to fatty liver), exclusion of excessive alcohol intake (ethanol intake: male: ≥ 210 g/week; female: ≥ 140 g/week), and the presence of at least one metabolic risk factor for cardiovascular disease: (1) Body mass index (BMI) ≥ 24.0 kg/m^2^ or waist circumference (WC) ≥ 90 cm (male) ≥ 85 cm (female) or body fat content and percentage body fat exceeded the standard; (2) Fasting plasma glucose (FPG) ≥ 6.1 mmol/L or 2-h post-load glucose levels ≥ 7.8 mmol/L or glycated hemoglobin ≥ 5.7% or diagnosis of type 2 diabetes or treatment for type 2 diabetes; (3) Blood pressure ≥ 130/85 mmHg or specific antihypertensive drug treatment; (4) Plasma triglycerides (TG) ≥ 1.70 mmol/L or lipid lowering treatment; (5) Plasma high-density lipoprotein cholesterol (HDL-C) ≤ 1.0 mmol/L (male) or ≤ 1.3 mmol/L (female) or lipid lowering treatment. These criteria are similar to the international MASLD diagnostic criteria, except that the thresholds of certain variables have been optimized according to the epidemiological characteristics of the Chinese population.

In addition, to enhance the comparability of the study findings, we also applied the diagnostic criteria from the International Multi-Society Consensus on MASLD (2023) (23).

### 2.3 Assessment of GNRI

Ideal body weight is calculated using the Lorentz formula. For males, ideal body weight = height (cm) – 100 – [(height (cm) – 150)/4.0]; for females, ideal body weight = height (cm) – 100 – [(height (cm) – 150)/2.5]. The formula for calculating GNRI: GNRI = [1.489 × serum albumin value (g/L)] + [41.7 × actual body weight (kg)/ideal body weight (kg)]. To ensure the weight of albumin and to mitigate the effect of disease states (oedema, pleural, and abdominal effusions) on body weight, in the GNRI formula, if actual body weight > ideal body weight, actual body weight/ideal body weight = 1; if actual body weight < ideal body weight, the calculation is based on the actual ratio. Grouping was based on GNRI quartiles (Q1–Q4), with GNRI < 104.79 in group Q1, 104.79 ≤ GNRI < 107.51 in group Q2, 107.51 ≤ GNRI < 109.77 in group Q3, and GNRI ≥ 109.77 in group Q4. Furthermore, we also employed four nutrition-related risk levels as defined in previous study: high risk (GNRI < 82), medium risk (82 ≤ GNRI < 92), low risk (92 ≤ GNRI ≤ 98), and no risk (GNRI > 98) (14).

### 2.4 Other definitions and measurements

Age was classified into two groups: age < 75 and age ≥ 75. Height, weight, and WC were measured by investigators who had received uniform training, using standard measurement methods. BMI was calculated as weight in kilograms divided by height in meters squared, was categorized as two groups: BMI < 24.0 kg/m^2^ and BMI ≥ 24.0 kg/m^2^. Ethnic group was categorized as Han ethnicity and ethnic minorities. Educational attainment was classified as primary school and below, junior high school, high school and undergraduate and above. Marital status was categorized into four groups: unmarried, married or cohabiting, divorced or separated, and widowed. Participants were classified as never smokers (individuals who had never smoked in their lives), former smokers (those who had smoked ≥ 100 cigarettes but had quit), and current smokers (those who had smoked ≥ 100 cigarettes and were still smoking). Regular drinkers were defined as individuals who consumed alcoholic beverages at least once a week for a continuous period of six months. Those who did not meet this criterion were defined as non-drinkers. After fasting for at least 8 h, fasting venous blood samples were collected from each study participant by professional medical workers. The collected blood samples were stored in a low-temperature transport box and sent to the hospital's clinical laboratory within 2 h for biochemical parameter tests. The biochemical parameters included fasting plasma glucose, total cholesterol (TC), TG, low-density lipoprotein cholesterol (LDL-C), HDL-C, alanine aminotransferase (ALT), aspartate aminotransferase (AST), and serum uric acid (UA). Hypertensive patients were defined as those who met at least one of the following criteria: self-reported hypertension, systolic blood pressure ≥ 140 mmHg, diastolic blood pressure ≥ 90 mmHg, hypertension documented in medical records, or receiving antihypertensive treatment. Glycemic status was defined as a three-category variable (normal, pre-diabetes, diabetes). Diabetic patients were defined as those who met at least one of the following criteria: self-reported diabetes, FPG ≥ 7.0 mmol/L, glycated hemoglobin (HbA1c) ≥ 6.5%, diabetes documented in medical records, or receiving antidiabetic treatment. Pre-diabetes was defined as meeting laboratory criteria (FPG 5.6–6.9 mmol/L or HbA1c 5.7–6.4%) in the absence of diabetes diagnosis or treatment. Normal glycemic status was defined as FPG < 5.6 mmol/L and HbA1c < 5.7% with no evidence of diabetes. This study categorized patients with MASLD into three subtypes. Individuals diagnosed with diabetes are classified as MASLD (diabetes subtype), while among those without diabetes, participants with a BMI ≥ 24 kg/m^2^ [according to Chinese BMI standards (24)] are considered to have MASLD (overweight/obesity subtype). Finally, in the non-diabetic population, individuals with a BMI < 24 kg/m^2^ but with metabolic risk abnormalities are classified as MASLD (lean metabolic disorder subtype), while the remaining individuals are classified as non-MASLD (9). In parallel, an international classification was applied using the Asian-specific BMI cut-off of ≥ 23 kg/m^2^, as specified in the consensus MASLD diagnostic criteria (2023) (23). Within this framework, non-diabetic patients with BMI ≥ 23 kg/m^2^ were categorized into the overweight/obesity subtype, and those with BMI < 23 kg/m^2^ but with accompanying cardio metabolic abnormalities were assigned to the lean metabolic disorder subtype.

### 2.5 Statistical analysis

Continuous variables that conformed to normal distribution were described by mean ± standard deviation and were compared between groups using the independent samples *t*-test; continuous variables that did not conform to normal distribution were expressed as median and upper and lower quartiles [M (P_25_, P_75_)], and comparisons between groups were made using a nonparametric test (Mann-Whitney test). Categorical variables were described by frequency and percentage, while the differences among groups were compared using the chi-square test. To explore the independent association between GNRI and the risk of MASLD in the elderly population, and to estimate the odds ratios (ORs) and 95% confidence intervals (CIs), multivariate binary logistic regression models were employed. Three models were incorporated to control for confounders. The included covariates are based on previous study covering MASLD, aging, and nutrition (20, 21, 25–31). Crude model was non-adjusted. Model 1 was adjusted for gender, age group, ethnic group, marital status and educational attainment, model 2 was further adjusted for smoking status, drinking status, BMI group and WC, and model 3 was further adjusted for ALT, AST, UA, TC, TG, HDL-C, hypertension and glycemic status. All the values of variance inflation factors in the final model were below five, confirming the absence of multicollinearity. To investigate whether there was a nonlinear association between GNRI and the risk of MASLD in the elderly population, restricted cubic splines (RCS) (3 knots: at the 10th, 50th, and 90th percentiles) and likelihood ratio test were conducted. The selection of knots for the RCS curves was guided by the minimization of Akaike's Information Criterion. If the association was nonlinear, the inflection point was determined by identifying the GNRI value at which the predicted OR from the model equaled 1. The receiver operator characteristic curve (ROC) and the area under the curve (AUC) were employed to access the predictive accuracy of multivariate-adjusted GNRI for the risk of MASLD. Mediation analyses were used to investigate whether the relevance of GNRI to MASLD could be explained by BMI, WC, ALT, AST, TC, TG, LDL-C, and HDL-C after adjusting for covariates in the model 3. Stratified analyses were conducted based on gender, age, BMI, smoking status, drinking status, hypertension, and glycemic status. Additionally, we used multinomial logistic regression with multivariable adjustment to further explore whether an increase in GNRI (or higher quantiles) would increase the risk of a particular MASLD subtype compared to individuals without MASLD. Three models were included in the study to control for confounding factors. The crude model was unadjusted. Model 1 adjusted for gender, age group, ethnic group, marital status, and educational attainment. Model 2 further adjusted for smoking status and drinking status. Model 3 further adjusted for ALT, AST, UA, TC, TG, HDL-C, and hypertension. All logistic regression models were developed in adherence to the widely accepted criterion of a minimum of 10 outcome events per predictor variable. In this study, several variables (ethnic group, marital status, and educational attainment) exhibited some degree of missingness (11.55%, 11.06%, and 10.45%, respectively). For all other analytical variables, the missing rates were below 1% or the data were complete. All missing data were imputed with multiple imputation methods.

We conducted additional analyses to examine whether GNRI is associated with the risk of MASLD (as defined by the 2023 International Multi-Society Consensus) and its subtypes in Chinese elderly individuals. We also added a full analysis of the international MASLD criteria, and the results of this part are presented in the supplementary material.

All statistical analyses were conducted using SAS version 9.4 (SAS Institute, Cary, NC) and R version 4.3.2 (R Foundation for Statistical Computing, Vienna, Austria). A two-sided *P* value < 0.05 was regarded as statistically significant.

## 3 Results

### 3.1 Baseline characteristics of study participants

Among the 7,628 participants, 40.4% were male and 59.6% were female, and the prevalence of MASLD was 38.8% (2,956 cases). The median age of the participants was 67 years, and the median BMI was 23.82 kg/m^2^. The characteristics of individuals with MASLD or non-MASLD are summarized in Table 1. According to the GNRI nutritional risk classification, 96.6% of all participants in this study were classified as no risk, including 99.6% of those with MASLD and 94.7% of those without MASLD. Individuals with MASLD were more likely to be aged < 75 years, female, married or cohabiting, never smokers, non-drinkers, BMI > 24.0 kg/m^2^. Compared with those who were without MASLD, participants with MASLD tended to have a higher GNRI, BMI, WC, FPG, ALT, AST, UA, TC, TG, LDL-C, and a lower HDL-C. Additionally, those with MASLD had a higher prevalence of hypertension and diabetes.

**Table 1:** Characteristics among individuals with MASLD or non-MASLD.

**Characteristics**	**Total (*n* = 7,628)**	**MASLD (*n* = 2,956)**	**Non-MASLD (*n* = 4,672)**	***P* value**
Age (year)	67.00 (64.00, 71.00)	67.00 (64.00, 71.00)	67.00 (64.00, 72.00)	< 0.001
**Age group (year)**				
< 75	6,593 (86.4%)	2,648 (89.6%)	3,945 (84.4%)	< 0.001
≥ 75	1,035 (13.6%)	308 (10.4%)	727 (15.6%)	
**Gender**				
Male	3,080 (40.4%)	971 (32.8%)	2,109 (45.1%)	< 0.001
Female	4,548 (59.6%)	1,985 (67.2%)	2,563 (54.9%)	
**Ethnic group**				
Han ethnicity	7,610 (99.8%)	2,946 (99.7%)	4,664 (99.8%)	0.143
Ethnic minorities	18 (0.2%)	10 (0.3%)	8 (0.2%)	
**Educational attainment**				
Primary school and below	3,954 (51.8%)	1,565 (52.9%)	2,389 (51.1%)	0.437
Junior high school	2,105 (27.6%)	793 (26.8%)	1,312 (28.1%)	
Senior high school	1,218 (16.0%)	460 (15.6%)	758 (16.2%)	
Undergraduate and above	351 (4.6%)	138 (4.7%)	213 (4.6%)	
**Marital status**				
Unmarried	158 (2.1%)	85 (2.9%)	73 (1.6%)	< 0.001
Married or cohabiting	6,353 (83.3%)	2,449 (82.8%)	3,904 (83.6%)	
Divorced or separated	130 (1.7%)	64 (2.2%)	66 (1.4%)	
Widowed	987 (12.9%)	358 (12.1%)	629 (13.5%)	
**Smoking status**				
Never smokers	6,187 (81.1%)	2,543 (86.0%)	3,644 (78.0%)	< 0.001
Current smokers	827 (10.8%)	219 (7.4%)	608 (13.0%)	
Former smokers	614 (8.1%)	194 (6.6%)	420 (9.0%)	
**Drinking status**				
Non-drinkers	6,961 (91.3%)	2,796 (94.6%)	4,165 (89.1%)	< 0.001
Regular drinkers	667 (8.7%)	160 (5.4%)	507 (10.9%)	
BMI (kg/m^2^)	23.82 (21.85, 25.99)	25.11 (23.19, 27.23)	23.04 (21.09, 25.05)	< 0.001
**BMI group (kg/m** ^2^ **)**				
< 24.0	4,001 (52.5%)	1,047 (35.4%)	2,954 (63.2%)	< 0.001
≥24.0	3,627 (47.5%)	1,909 (64.6%)	1,718 (36.8%)	
WC (cm)	86.30 (80.20, 92.20)	89.60 (84.00, 95.50)	84.15 (78.00, 89.90)	< 0.001
FPG (mmol/L)	5.36 (4.92, 6.00)	5.60 (5.10, 6.53)	5.22 (4.86, 5.75)	< 0.001
ALT (U/L)	18.30 (14.20, 25.00)	21.30 (16.13, 30.10)	16.85 (13.30, 22.10)	< 0.001
AST (U/L)	22.20 (19.30, 26.30)	23.05 (19.70, 27.70)	21.80 (19.10, 25.48)	< 0.001
TC (mmol/L)	5.37 (4.67, 6.12)	5.49 (4.74, 6.25)	5.31 (4.63, 6.05)	< 0.001
TG (mmol/L)	1.46 (1.06, 2.08)	1.77 (1.30, 2.47)	1.30 (0.96, 1.79)	< 0.001
LDL-C (mmol/L)	3.06 (2.51, 3.61)	3.16 (2.61, 3.74)	3.00 (2.47, 3.53)	< 0.001
HDL-C (mmol/L)	1.28 (1.09, 1.50)	1.21 (1.05, 1.40)	1.33 (1.13, 1.56)	< 0.001
UA (μmol/L)	347.90 (288.90, 417.00)	365.95 (307.00, 436.00)	333.35 (279.63, 401.38)	< 0.001
**Hypertension**				
No	2,948 (38.6%)	987 (33.4%)	1,961 (42.0%)	< 0.001
Yes	4,680 (61.4%)	1,969 (66.6%)	2,711 (58.0%)	
**Glycemic status**				
Normal	3,956 (51.9%)	1,045 (35.4%)	2,911 (62.3%)	< 0.001
Pre-diabetes	1,959 (25.7%)	967 (32.7%)	992 (21.2%)	
Diabetes	1,713 (22.5%)	944 (31.9%)	769 (16.5%)	
GNRI	107.51 (104.79, 109.77)	108.71 (106.51, 110.79)	106.62 (103.67, 109.00)	< 0.001
**GNRI grades of nutrition-related risk**				
Major risk	4 (0.1%)	0 (0.0%)	4 (0.1%)	< 0.001
Moderate risk	26 (0.3%)	0 (0.0%)	26 (0.6%)	
Low risk	229 (3.0%)	12 (0.4%)	217 (4.6%)	
No risk	7,369 (96.6%)	2,994 (99.6%)	4,425 (94.7%)	
**GNRI quartiles group**				
Q1	1,907 (25.0%)	356 (12.0%)	1,551 (33.2%)	< 0.001
Q2	1,857 (24.3%)	662 (22.4%)	1,195 (25.6%)	
Q3	1,956 (25.6%)	865 (29.3%)	1,091 (23.4%)	
Q4	1,908 (25.0%)	1,073 (36.3%)	835 (17.9%)	

Data are presented as median (P_25_, P_75_) for continuous variables and frequency (percentages) for categorical variables.

ALT, alanine aminotransferase; AST, aspartate aminotransferase; BMI, body mass index; GNRI, geriatric nutritional risk index; HDL-C, high-density lipoprotein cholesterol; LDL-C, low-density lipoprotein cholesterol; MASLD, metabolic dysfunction-associated steatotic liver disease; TC, total cholesterol; TG, triglycerides; OR, odds ratio; Q, quartile; WC, waist circumference; UA, uric acid.

### 3.2 Association between GNRI and the risk of MASLD in the elderly population

Table 2 presents the multivariate binary logistic regression analysis of GNRI and the risk of MASLD. In Model 3, after adjusting for potential confounding factors, each 1-unit increase in GNRI was associated with a 12% higher risk of MASLD (OR = 1.12, 95% CI: 1.10–1.13). Similarly, when GNRI was categorized into quartiles, compared with the first quartile (Q1), the higher the GNRI level, the higher the prevalence of MASLD in the elderly population. After full adjustment, the ORs and 95% CIs for GNRI quartiles and the risk of MASLD were as follows: Q2: OR = 1.56, 95% CI: 1.31–1.84; Q3: OR = 1.93, 95% CI: 1.64–2.28; Q4: OR = 2.70, 95% CI: 2.28–3.20; *P* for trend < 0.001. Collectively, these results indicate a dose-response relationship, where both per-unit increases and higher quartiles of GNRI consistently elevate MASLD risk.

**Table 2:** Association between the GNRI and MASLD based on binary logistic regression analysis.

**Model**	**GNRI continuous OR (95% CI)**	GNRI Quartiles OR (95% CI)				***P* for trend**
		**Q1**	**Q2**	**Q3**	**Q4**	
Crude^a^	1.19 (1.17, 1.20)	1.00	2.41 (2.08, 2.80)	3.45 (2.99, 4.00)	5.60 (4.84, 6.48)	< 0.001
Model 1^b^	1.18 (1.17, 1.20)	1.00	2.30 (1.98, 2.68)	3.25 (2.81, 3.77)	5.38 (4.64, 6.24)	< 0.001
Model 2^c^	1.14 (1.12, 1.16)	1.00	1.73 (1.48, 2.03)	2.22 (1.90, 2.60)	3.60 (3.08, 4.21)	< 0.001
Model 3^d^	1.12 (1.10, 1.13)	1.00	1.56 (1.31, 1.84)	1.93 (1.64, 2.28)	2.70 (2.28, 3.20)	< 0.001

^a^Non-adjusted. ^b^Adjusted for gender, age group, ethnic group, marital status and educational attainment. ^c^Further adjusted for smoking status, drinking status, BMI group and WC. ^d^Further adjusted for ALT, AST, UA, TC, TG, HDL-C, hypertension and glycemic status.

ALT, alanine aminotransferase; AST, aspartate aminotransferase; BMI, body mass index; CI, confidence interval; GNRI, geriatric nutritional risk index; HDL-C, high-density lipoprotein cholesterol; MASLD, metabolic dysfunction-associated steatotic liver disease; TC, total cholesterol; TG, triglycerides; OR, odds ratio; Q, quartile; WC, waist circumference; UA, uric acid.

### 3.3 Nonlinear trends of GNRI with MASLD in the elderly population

**Figure 2:**
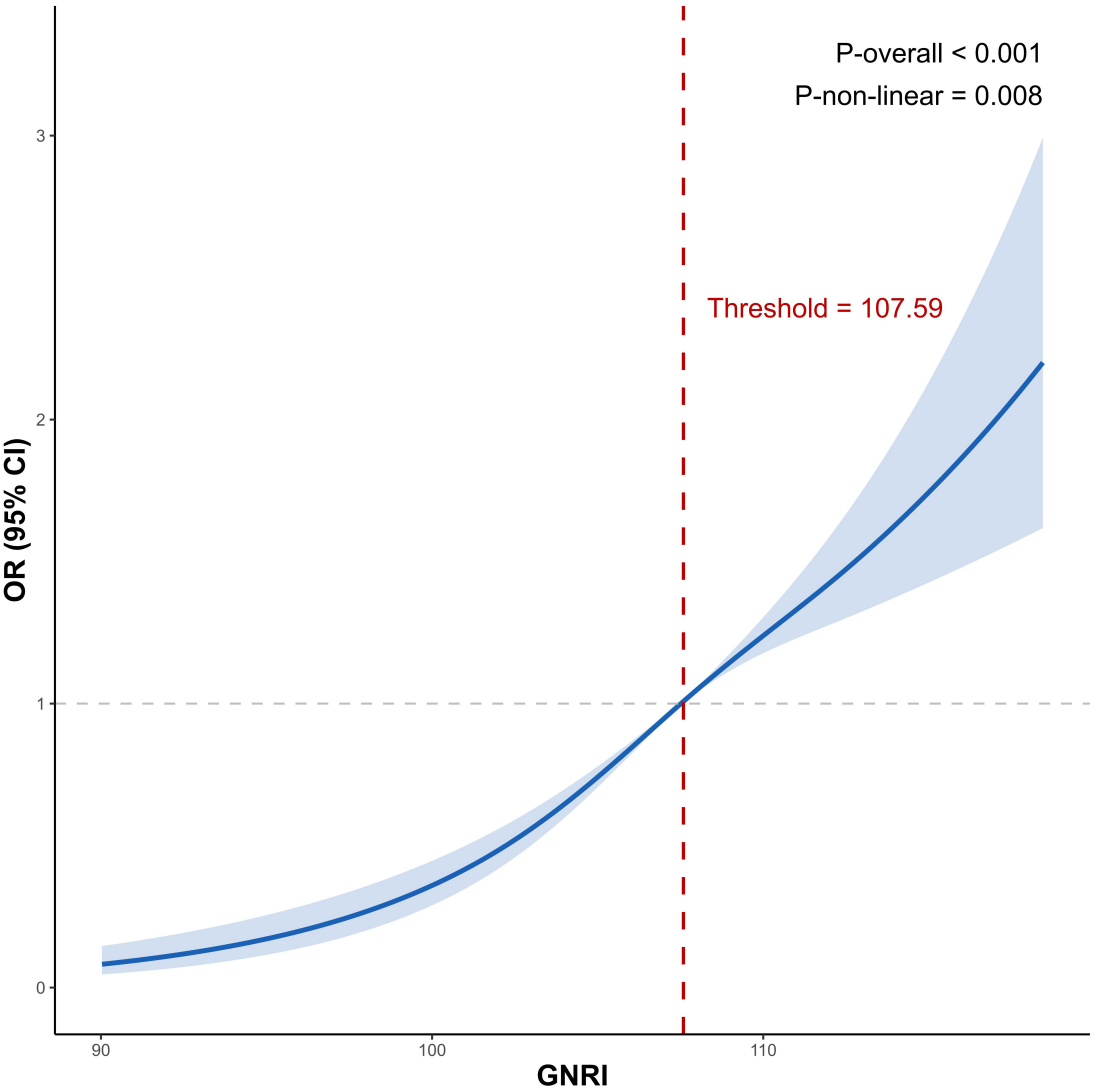
Nonlinear association between GNRI and MASLD risk by restricted cubic spline analysis. The y-axis represented the OR and 95% CI for MASLD, while the x-axis indicated the GNRI. The solid line and blue area represented the estimate and corresponding 95% CI. GNRI = 107.59 was used as the reference point (OR = 1). Adjusted for gender, age group, marital status, educational attainment, smoking status, drinking status, BMI group, WC, ALT, AST, UA, TC, TG, HDL-C, hypertension and glycemic status. ALT, alanine aminotransferase; AST, aspartate aminotransferase; BMI, body mass index; CI, confidence interval; GNRI, geriatric nutritional risk index; HDL-C, high-density lipoprotein cholesterol; MASLD, metabolic dysfunction-associated steatotic liver disease; OR, odds ratio; TC, total cholesterol; TG, triglycerides; WC, waist circumference; UA, uric acid.

To investigate whether there was a nonlinear association between GNRI and the prevalence of MASLD, RCS, and likelihood ratio test were conducted (Figure 2). The results demonstrated a nonlinear J-shaped association between GNRI and the risk of MASLD (*P* for overall < 0.001; *P* for nonlinear = 0.008). Taking GNRI = 107.59 as the reference point (OR = 1), we observed that: when GNRI < 107.59, the OR for MASLD was consistently < 1, suggesting a protective effect. When GNRI ≥ 107.59, the OR increased progressively, indicating an increased risk.

### 3.4 Predictive value of GNRI for MASLD in the elderly population

**Figure 3:**
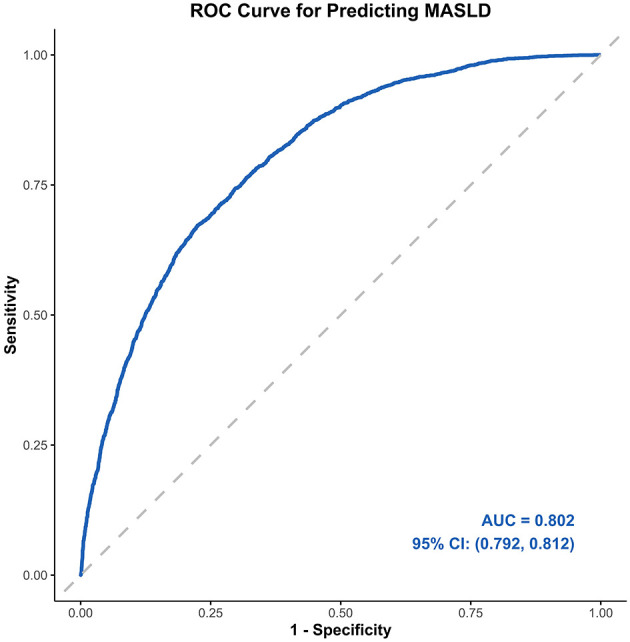
Receiver operator characteristic curve of GNRI for predicting MASLD. The figure shows the predictive effect of GNRI on the risk of MASLD. Adjusted for gender, age group, marital status, educational attainment, smoking status, drinking status, BMI group, WC, ALT, AST, UA, TC, TG, HDL-C, hypertension and glycemic status. ALT, alanine aminotransferase; AST, aspartate aminotransferase; AUC, area under the curve; BMI, body mass index; CI, confidence interval; GNRI, geriatric nutritional risk index; HDL-C, high-density lipoprotein cholesterol; MASLD, metabolic dysfunction-associated steatotic liver disease; ROC, receiver operating characteristic curve; TC, total cholesterol; TG, triglycerides; WC, waist circumference; UA, uric acid.

The ROC and AUC were employed to assess the predictive accuracy of multivariate-adjusted GNRI for the risk of MASLD (Figure 3). The GNRI demonstrated predictive capability for the prevalence of MASLD in the elderly population (AUC: 0.802, 95% CI: 0.792–0.812).

### 3.5 Mediation analysis of GNRI and MASLD

**Figure 4:**
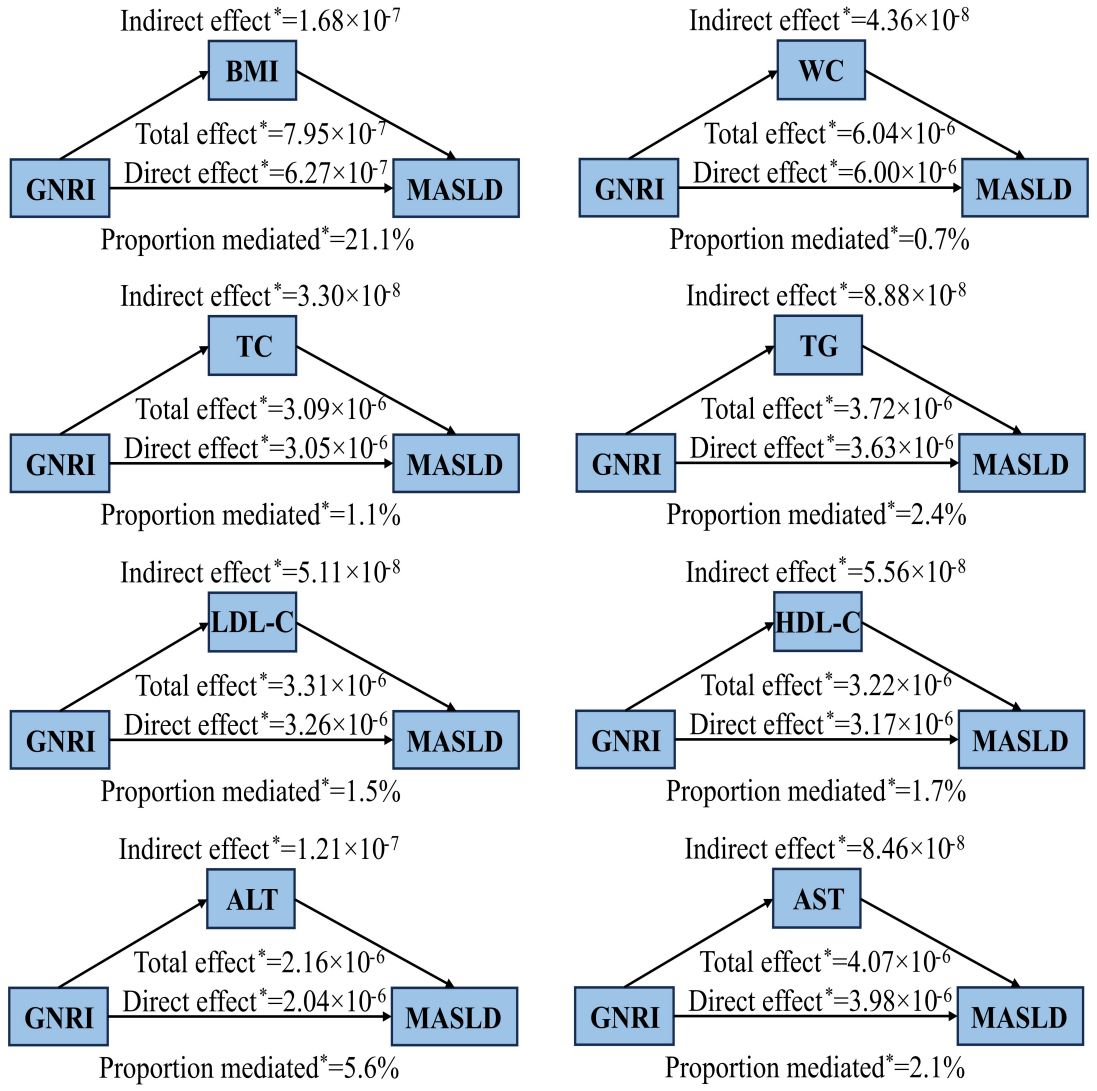
Mediation analysis for the associations between GNRI and MASLD in the elderly population. ^*^P < 0.05. Adjusted for gender, age group, marital status, educational attainment, smoking status, drinking status, BMI group, WC, ALT, AST, UA, TC, TG, HDL-C, hypertension and glycemic status. In the process of mediation analysis, the mediator variables are excluded from the adjustment to accurately estimate the direct and indirect effects between the GNRI and MASLD. ALT, alanine aminotransferase; AST, aspartate aminotransferase; BMI, body mass index; GNRI, geriatric nutritional risk index; HDL-C, high-density lipoprotein cholesterol; LDL-C, low-density lipoprotein cholesterol; MASLD, metabolic dysfunction-associated steatotic liver disease; TC, total cholesterol; TG, triglycerides; WC, waist circumference; UA, uric acid.

Mediation analyses indicated that BMI, WC, TC, TG, LDL-C, HDL-C, ALT, and AST significantly mediated the association between GNRI and MASLD (Figure 4). Among the examined mediators, BMI accounted for the largest proportion of the total effect of GNRI on MASLD (21.1%, 95% CI: 6.4%−29.2%), followed by ALT (5.6%), TG (2.4%), AST (2.1%), HDL-C (1.7%), LDL-C (1.5%), TC (1.1%), and WC (0.7%), all of which exhibited statistically significant mediation effects (*P* < 0.05).

### 3.6 Stratified analyses

After adjusting for potential confounders, stratified analyses based on gender, age, BMI, smoking status, drinking status, hypertension, and glycemic status demonstrated that the risk of MASLD increased with increasing levels of the GNRI quartile for people with different characteristics (Table 3). Among them, male with high GNRI levels, those aged ≥ 75 years, those with a BMI < 24.0 kg/m^2^, current smokers, regular drinkers, people with hypertension and normal glycemic status have a relatively higher risk of MASLD.

**Table 3:** Stratified analysis of the association between the GNRI and MASLD.

**Characteristics**	**GNRI continuous OR (95% CI)**	GNRI Quartiles OR (95% CI)				***P* for trend**
		**Q1**	**Q2**	**Q3**	**Q4**	
**Gender**			
Male	1.13 (1.10, 1.15)	1.00	1.81 (1.36, 2.40)	2.39 (1.80, 3.17)	3.38 (2.56, 4.45)	< 0.001
Female	1.10 (1.07, 1.12)	1.00	1.42 (1.15, 1.76)	1.69 (1.37, 2.08)	2.30 (1.86, 2.86)	< 0.001
**Age (year)**			
< 75	1.11 (1.09, 1.13)	1.00	1.51 (1.26, 1.82)	1.88 (1.57, 2.25)	2.65 (2.21, 3.18)	< 0.001
≥75	1.12 (1.07, 1.17)	1.00	1.73 (1.12, 2.69)	2.18 (1.42, 3.36)	3.03 (1.87, 4.91)	< 0.001
**BMI (kg/m** ^2^ **)**			
< 24.0	1.14 (1.11, 1.16)	1.00	2.08 (1.63, 2.66)	2.47 (1.92, 3.17)	3.64 (2.82, 4.69)	< 0.001
≥24.0	1.09 (1.06, 1.11)	1.00	1.16 (0.91, 1.48)	1.46 (1.15, 1.85)	2.00 (1.57, 2.53)	< 0.001
**Smoking status**			
Never smokers	1.11 (1.09, 1.13)	1.00	1.57 (1.30, 1.89)	1.91 (1.59, 2.29)	2.61 (2.17, 3.15)	< 0.001
Current smokers	1.14 (1.09, 1.20)	1.00	1.79 (1.03, 3.10)	2.54 (1.43, 4.49)	3.63 (2.13, 6.17)	< 0.001
Former smokers	1.1 (1.04, 1.17)	1.00	1.27 (0.66, 2.41)	1.70 (0.93, 3.11)	2.45 (1.33, 4.53)	0.002
**Drinking status**			
Non-drinkers	1.11 (1.09, 1.13)	1.00	1.52 (1.27, 1.81)	1.84 (1.54, 2.18)	2.60(2.18, 3.10)	< 0.001
Regular drinkers	1.11 (1.05, 1.18)	1.00	2.30 (1.13, 4.67)	3.29 (1.66, 6.53)	3.85 (1.94, 7.66)	< 0.001
**Hypertension**			
No	1.10 (1.08, 1.13)	1.00	1.62 (1.23, 2.13)	1.85 (1.41, 2.44)	2.46 (1.86, 3.25)	< 0.001
Yes	1.11 (1.09, 1.14)	1.00	1.51 (1.22, 1.88)	1.94 (1.57, 2.40)	2.84 (2.30, 3.51)	< 0.001
**Glycemic status**			
Normal	1.14 (1.11, 1.17)	1.00	1.56 (1.22, 1.99)	1.85 (1.45, 2.35)	3.24 (2.52, 4.18)	< 0.001
Pre-diabetes	1.09 (1.06, 1.12)	1.00	1.62 (1.15, 2.28)	1.96 (1.40, 2.73)	2.41 (1.74, 3.34)	< 0.001
Diabetes	1.09 (1.05, 1.12)	1.00	1.45 (1.05, 2.01)	2.05 (1.47, 2.85)	2.25 (1.64, 3.09)	< 0.001

Adjusted for gender, age group, marital status, educational attainment, smoking status, drinking status, BMI group, WC, ALT, AST, UA, TC, TG, HDL-C, hypertension, and glycemic status. The strata variable was not included when stratifying by itself.

ALT, alanine aminotransferase; AST, aspartate aminotransferase; BMI, body mass index; CI, confidence interval; GNRI, geriatric nutritional risk index; HDL-C, high-density lipoprotein cholesterol; MASLD, metabolic dysfunction-associated steatotic liver disease; TC, total cholesterol; TG, triglycerides; OR, odds ratio; Q, quartile; WC, waist circumference; UA, uric acid.

### 3.7 Association of GNRI with MASLD subtypes compared with the Non-MASLD group

The ORs and 95% CIs for the association between GNRI and MASLD subtypes, estimated using multinomial logistic regression with multivariable adjustment and the non-MASLD group as the reference, are presented in Table 4. Regardless of the MASLD subtype, higher GNRI levels (or higher quantiles) were significantly associated with an increased risk of that subtype compared with individuals without MASLD. When GNRI was categorized into quartiles, the risk of belonging to the overweight/obese subtype was the highest, followed by the diabetic subtype and then the lean metabolic disorder subtype. Similar trends were observed when GNRI was analyzed as a continuous variable. After adjusting for covariates in model 3, when the GNRI Q1 group was set as the reference, the ORs and 95% CIs were as follows: MASLD (diabetes): Q2: OR = 1.54, 95% CI: 1.20–1.97; Q3: OR = 2.08, 95% CI: 1.64–2.64; Q4: OR = 3.36, 95% CI: 2.66–4.25; MASLD (overweight/obesity): Q2: OR = 2.21, 95% CI: 1.76–2.78; Q3: OR = 3.04, 95% CI: 2.43–3.79; Q4: OR = 4.90, 95% CI: 3.93–6.11; MASLD (lean metabolic disorder): Q2: OR = 1.82, 95% CI: 1.39–2.37; Q3: OR = 2.23, 95% CI: 1.72–2.89; Q4: OR = 3.04, 95% CI: 2.34–3.95; all *P*_−trend_ < 0.001.

**Table 4:** Multiple logistic regression analysis of the association between GNRI and MASLD subtypes using non-MASLD as the reference group.

**MASLD subtypes**	**GNRI continuous OR (95% CI)**	GNRI Quartiles OR (95% CI)				***P* for trend**
		**Q1**	**Q2**	**Q3**	**Q4**	
**MASLD (diabetes) (*****n*** = **944)**			
Crude^a^	1.18 (1.16, 1.20)	1.00	2.04 (1.62, 2.59)	2.99 (2.39, 3.74)	5.12 (4.11, 6.38)	< 0.001
Model 1^b^	1.18 (1.16, 1.20)	1.00	1.99 (1.57, 2.52)	2.89 (2.30, 3.62)	5.03 (4.03, 6.29)	< 0.001
Model 2^c^	1.18 (1.16, 1.20)	1.00	1.97 (1.56, 2.50)	2.87 (2.29, 3.61)	5.00 (4.00, 6.26)	< 0.001
Model 3^d^	1.14 (1.12, 1.16)	1.00	1.54 (1.20, 1.97)	2.08 (1.64, 2.64)	3.36 (2.66, 4.25)	< 0.001
**MASLD (overweight/obesity) (*****n*** = **1,299)**			
Crude^a^	1.21 (1.19, 1.23)	1.00	2.85 (2.28, 3.55)	4.24 (3.43, 5.25)	7.09 (5.74, 8.75)	< 0.001
Model 1^b^	1.21 (1.19, 1.23)	1.00	2.73 (2.18, 3.40)	4.01 (3.23, 4.97)	6.84 (5.53, 8.46)	< 0.001
Model 2^c^	1.21 (1.18, 1.23)	1.00	2.69 (2.16, 3.37)	3.94 (3.18, 4.89)	6.74 (5.44, 8.34)	< 0.001
Model 3^d^	1.17 (1.15, 1.20)	1.00	2.21 (1.76, 2.78)	3.04 (2.43, 3.79)	4.90 (3.93, 6.11)	< 0.001
**MASLD (lean metabolic disorder) (*****n*** = **713)**			
Crude^a^	1.15 (1.13, 1.18)	1.00	2.32 (1.79, 3.00)	3.02 (2.35, 3.88)	4.26 (3.32, 5.47)	< 0.001
Model 1^b^	1.15 (1.12, 1.17)	1.00	2.15 (1.66, 2.78)	2.73 (2.12, 3.53)	3.92 (3.04, 5.05)	< 0.001
Model 2^c^	1.15 (1.12, 1.17)	1.00	2.15 (1.66, 2.79)	2.75 (2.13, 3.55)	3.93 (3.05, 5.07)	< 0.001
Model 3^d^	1.12 (1.09, 1.15)	1.00	1.82 (1.39, 2.37)	2.23 (1.72, 2.89)	3.04 (2.34, 3.95)	< 0.001

^a^Non-adjusted. ^b^Adjusted for gender, age group, marital status, ethnic group and educational attainment. ^c^Further adjusted for smoking status and drinking status. ^d^Further adjusted for ALT, AST, UA, TC, TG, HDL-C and hypertension. Results are from multiple logistic regression analyses with non-MASLD as the reference group.

ALT, alanine aminotransferase; AST, aspartate aminotransferase; CI, confidence interval; GNRI, geriatric nutritional risk index; HDL-C, high-density lipoprotein cholesterol; MASLD, metabolic dysfunction-associated steatotic liver disease; TC, total cholesterol; TG, triglycerides; OR, odds ratio; Q, quartile; UA, uric acid.

## 4 Discussion

To our knowledge, this is the first comprehensive study examining the association between GNRI and the risk of MASLD and its subtypes in elderly Chinese (aged > 60 years). We observed a strong positive association between increasing GNRI levels and both overall MASLD risk and its subtypes. Critically, RCS analysis revealed a novel non-linear relationship between GNRI and MASLD risk. Further threshold analysis identified GNRI ≥ 107.59 as a critical point where MASLD risk significantly increased, highlighting the role of over nutrition. GNRI demonstrated relatively strong predictive ability for MASLD prevalence in this population. Mediation analyses indicated that BMI, WC, TC, TG, LDL-C, ALT, and AST significantly mediated the GNRI-MASLD association, with BMI explaining the largest proportion of the total effect. Furthermore, to enhance comparability, a parallel analysis using international diagnostic criteria for MASLD (2023) was conducted, yielding largely consistent findings.

At the 2023 annual meeting, the European Association for the Study of the Liver issued a new consensus recommending replacing metabolic dysfunction-associated fatty liver disease (MAFLD) with “MASLD” due to the negative connotations of “fatty” in the Western context (23). However, in China, where both terms are translated into Chinese, there is little difference between them, and the issue of negative connotations does not arise. Therefore, based on the latest scientific evidence, the Chinese Medical Association's Section of Hepatology has completely revised the 2018 edition of the guidelines and renamed them as the guidelines for the prevention and treatment of metabolic dysfunction-associated (non-alcoholic) fatty liver disease (2024 edition) (22). It clearly stipulates that the preferred English term is MAFLD, not MASLD, and that the two terms can be used interchangeably. Moreover, the new guideline takes into account the national context of clinical diagnosis and treatment in China. It proposes improved diagnostic criteria for MAFLD, with cardiovascular risk factors based on data from Chinese population studies, reflecting China's unique characteristics. As this study is also based on the Chinese population, the Chinese guidelines and diagnostic criteria were therefore applied.

The GNRI is a nutrition-related risk index that is widely used in clinical and epidemiological studies because of its simple calculation and easy availability of data (32). However, few studies have focused on the relationship between GNRI and MASLD risk in the Chinese elderly population. A Chinese cohort study found that the GNRI serve as a valuable predictor of the risk of NAFLD in non-obese individuals and that there was a linear relationship between the GNRI and NAFLD incidence (21). Another study, based on data from the 2017–2018 National Health and Nutrition Examination Survey, also observed a linear relationship between GNRI and NAFLD prevalence, but the association was not stable after adjusting for confounders (20). In our study, using the latest Chinese diagnostic criteria for MASLD and focusing on the elderly Chinese population (aged ≥ 60 years), a significant association was observed between higher GNRI levels and the prevalence of MASLD, which remained stable after adjusting for confounders, but the RCS curve showed a nonlinear relationship. This inconsistency with the findings of previous studies may be related to the composition of the study population, the sample size, the disease definition, the range of distribution of the GNRI, and the covariate adjustment strategy.

Initially used to identify nutrition-related risks, mainly those associated with nutritional deficiencies, in hospitalized elderly patients, GNRI has criteria for risk levels that have no upper limit, and thus a GNRI ≥ 98 indicates no nutritional risk without considering the risks posed by over nutrition (14). However, in our participants, 96.6% were at the no-nutritional-risk level. Similar GNRI distributions have also been reported in many studies, suggesting that most elderly people currently have no nutrition-related risks (21, 32–34). Using RCS curve, this study is the first to reveal a non-linear relationship between GNRI and the risk of MASLD. Further threshold analysis identified GNRI ≥ 107.59 as a critical point where MASLD risk significantly increased, highlighting the risk associated with over nutrition. Therefore, future efforts should re-evaluate GNRI's ability to identify health effects across the nutritional spectrum, encompassing not only the risk of malnutrition but also that of over nutrition, and establish a more suitable and practical risk classification standard. Our findings offer new evidence in support of this requirement.

Our study indicates that in the state of over nutrition, the elderly have a significantly increased risk of developing MASLD, which is also consistent with the results of the mediation analysis. The mediation analysis revealed that BMI, WC, TC, TG, LDL-C, HDL-C, ALT, and AST significantly mediated the association between GNRI and MASLD, with BMI accounting for the largest proportion of the total effect. This finding is consistent with extensive literature showing that increased BMI and central obesity are key drivers of hepatic steatosis and insulin resistance, which are central to the pathophysiology of MASLD (35–37). Previous studies have also demonstrated that WC, as a marker of visceral fat, is also strongly associated with MASLD (38, 39). Much evidence suggests that alterations in hepatic and extrahepatic lipid metabolism are central drivers of MASLD evolution (40). Dyslipidemia, characterized by elevated TC, TG, LDL-C, and decreased HDL-C, has been implicated in promoting liver fat accumulation and systemic inflammation (41, 42). Elevated liver enzymes such as ALT and AST are widely used clinical indicators of hepatic injury and are commonly elevated in patients with MASLD (43). Taken together, these findings suggest that the nutritional status captured by GNRI may influence MASLD risk primarily through metabolic and hepatic pathways. Our mediation analysis provides novel evidence supporting this hypothesis, especially in older adults, who may be more vulnerable to the combined effects of malnutrition and metabolic dysregulation. Therefore, GNRI may serve as a practical tool for reflecting nutritional and metabolic health status in the elderly population, which is beneficial for the early identification of high-risk individuals for MASLD.

This study further explored the association between GNRI and the risk of MASLD subtypes. Using the non-MASLD population as a reference, we found a significant and stable association between GNRI and the risk of the three different subtypes of MASLD. Moreover, the magnitude of the ORs demonstrated a gradient relationship: as GNRI levels increased, MASLD was most likely to be classified as the overweight/obesity subtype, followed by the diabetes subtype, and least likely as the lean metabolic disorder subtype. While it is generally accepted that over nutrition contributes to conditions like overweight, obesity, or diabetes, our analysis revealed that elevated GNRI also significantly associated with an increased risk of the lean metabolic disorder subtype of MASLD. This suggests that GNRI may be closely involved in the pathological mechanisms underlying this specific MASLD subtype. In the future, larger-scale cohort studies are required to further validate the ability of GNRI to identify MASLD and its subtypes and to deeply explore the mechanism underlying its association with lean metabolic disorder subtype of MASLD.

### 4.1 Strengths & limitations

This is a comprehensive study exploring the associations between GNRI and the risk of MASLD and its subtypes. We found that GNRI could be a strong predictor of MASLD risk in Chinese older adults using ROC analysis and RCS modeling—which revealed a significant non-linear relationship and identified GNRI ≥ 107.59 as a critical risk threshold. We also investigated the effect of mediating variables on the outcome using mediation analysis. In addition, we used the latest Chinese diagnostic criteria for MASLD and utilized ultrasonographic findings of hepatic steatosis to aid in the diagnosis. In order to enhance comparability, a parallel analysis using international diagnostic criteria for MASLD (2023) was conducted, yielding largely consistent findings. Furthermore, we rigorously accounted for potential confounders through multivariate adjustment across all analyses. Despite these strengths, several limitations warrant consideration. First, this is a cross-sectional study, so we cannot infer causal associations. Second, these results are based on older adults in China, which may limit replication and generalization to other populations. Third, although we attempted to control for confounding variables through multifactorial adjustment and stratified analyses, there may be other residual confounders. Finally, while GNRI was originally developed for hospitalized older adults, numerous studies have extended its application to community-dwelling elderly populations and those with specific chronic conditions, aligning with our approach. Nevertheless, further validation of GNRI for quantifying over nutrition-related risks (as opposed to its initial undernutrition focus) remains valuable.

## 5 Conclusions

This study demonstrates that elevated GNRI significantly increases MASLD and subtype risks in Chinese elderly (≥ 60 years). Critically, RCS analysis identified GNRI ≥107.59 as a threshold for substantially escalated the risk of MASLD. These findings support extending GNRI's application from traditional malnutrition assessment to over nutrition risk stratification. Integrating this threshold into prevention strategies could optimize metabolic hazard screening. Future studies should validate its clinical utility and explore nutritional transition mechanisms in MASLD pathogenesis.

## Data Availability

The (de-identified) raw data supporting the conclusions of this article will be made available by the authors upon reasonable request.
