# Supplementary material for: Association of geriatric nutritional risk index with metabolic dysfunction-associated steatotic liver disease and subtypes in Chinese elderly: identification of an overnutrition risk threshold and implications for extended risk stratification
Source: Front Nutr. 2026 Feb 13;13:1743679. doi: 10.3389/fnut.2026.1743679 (PMC12945829; doi:10.3389/fnut.2026.1743679)
Supplement: Supplementary file 1 [file Table_1.docx]

Supplementary Material

# Supplementary Figures and Tables

## Supplementary Figures


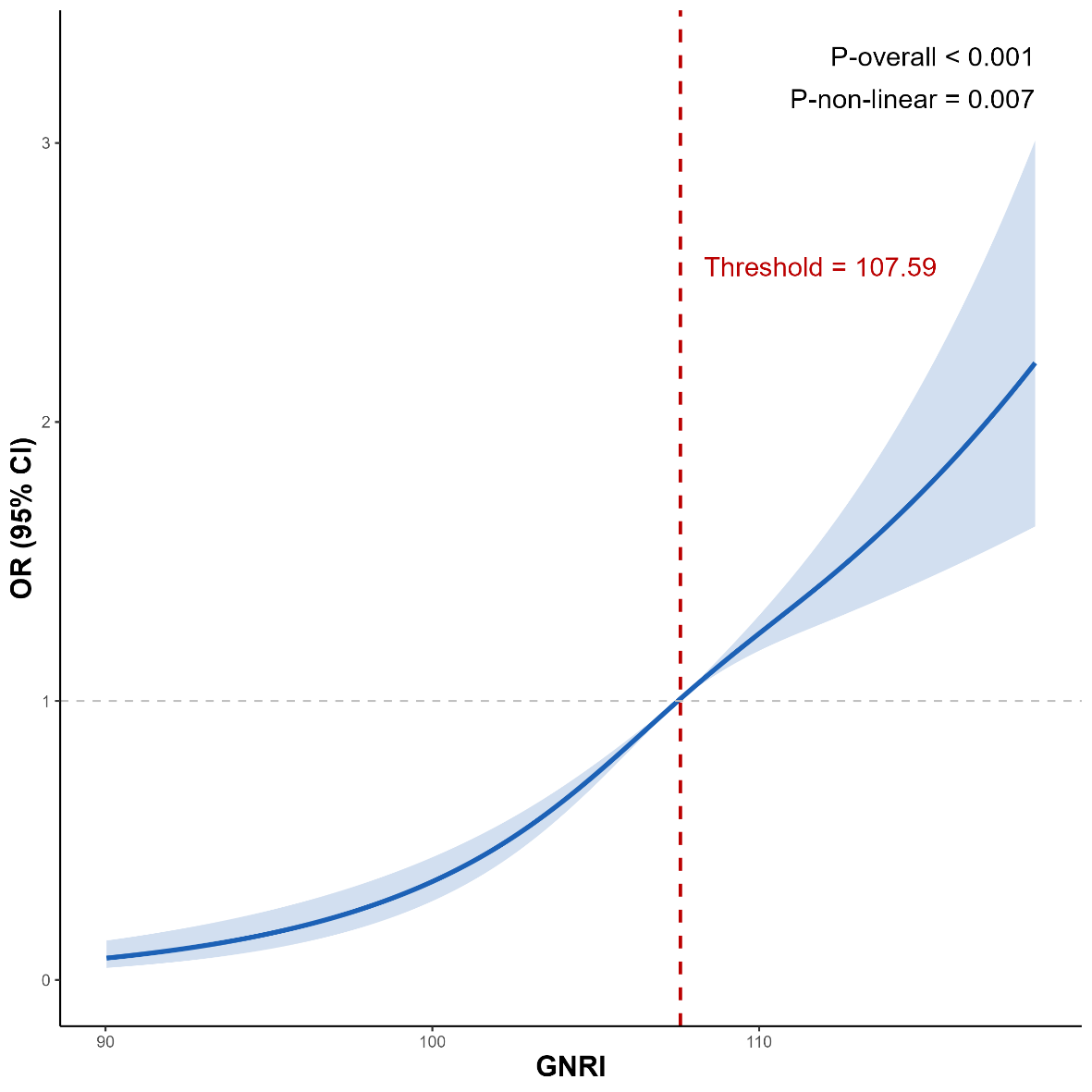


**Supplementary Figure 1.** **Nonlinear association between GNRI and MASLD** **(2023 International Multi-Society Consensus on MASLD) risk by restricted cubic spline analysis.** The y-axis represented the OR and 95% CI for MASLD, while the x-axis indicated the GNRI. The solid line and blue area represented the estimate and corresponding 95% CI. GNRI = 107.59 was used as the reference point (OR=1). Adjusted for gender, age group, marital status, educational attainment, smoking status, drinking status, BMI group, WC, ALT, AST, UA, TC, TG, HDL-C, hypertension and glycemic status. Abbreviations: ALT, alanine aminotransferase; AST, aspartate aminotransferase; BMI, body mass index; CI, confidence interval; GNRI, geriatric nutritional risk index; HDL-C, high-density lipoprotein cholesterol; MASLD, metabolic dysfunction-associated steatotic liver disease; OR, odds ratio; TC, total cholesterol; TG, triglycerides; WC, waist circumference; UA, uric acid.

**
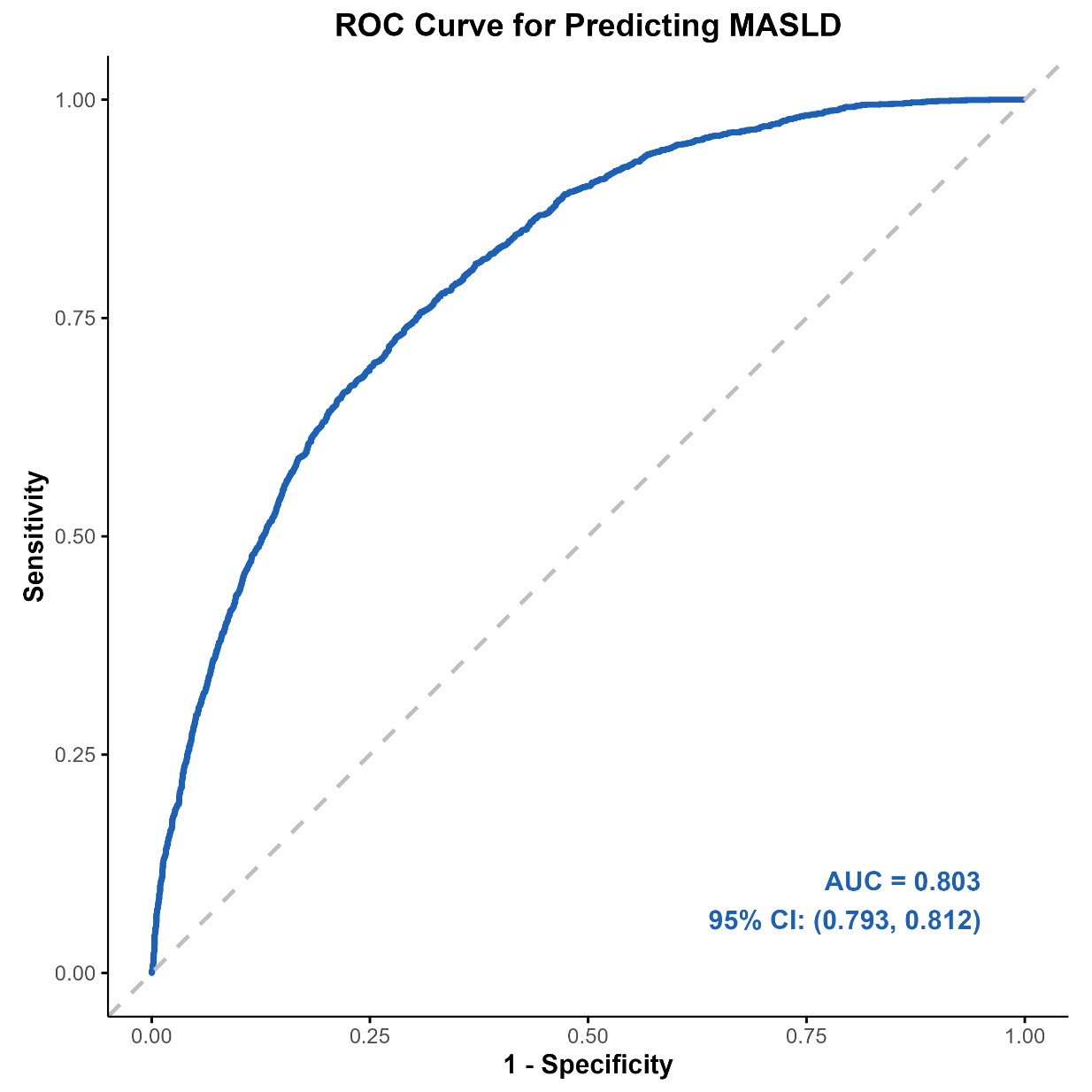
**

**Supplementary Figure 2. Receiver operator characteristic curve of GNRI for predicting MASLD (2023 International Multi-Society Consensus on MASLD).** The figure shows the predictive effect of GNRI on the risk of MASLD. Adjusted for gender, age group, marital status, educational attainment, smoking status, drinking status, BMI group, WC, ALT, AST, UA, TC, TG, HDL-C, hypertension and glycemic status. Abbreviations: ALT, alanine aminotransferase; AST, aspartate aminotransferase; AUC, area under the curve; BMI, body mass index; CI, confidence interval; GNRI, geriatric nutritional risk index; HDL-C, high-density lipoprotein cholesterol; MASLD, metabolic dysfunction-associated steatotic liver disease; ROC, receiver operating characteristic curve; TC, total cholesterol; TG, triglycerides; WC, waist circumference; UA, uric acid.

**
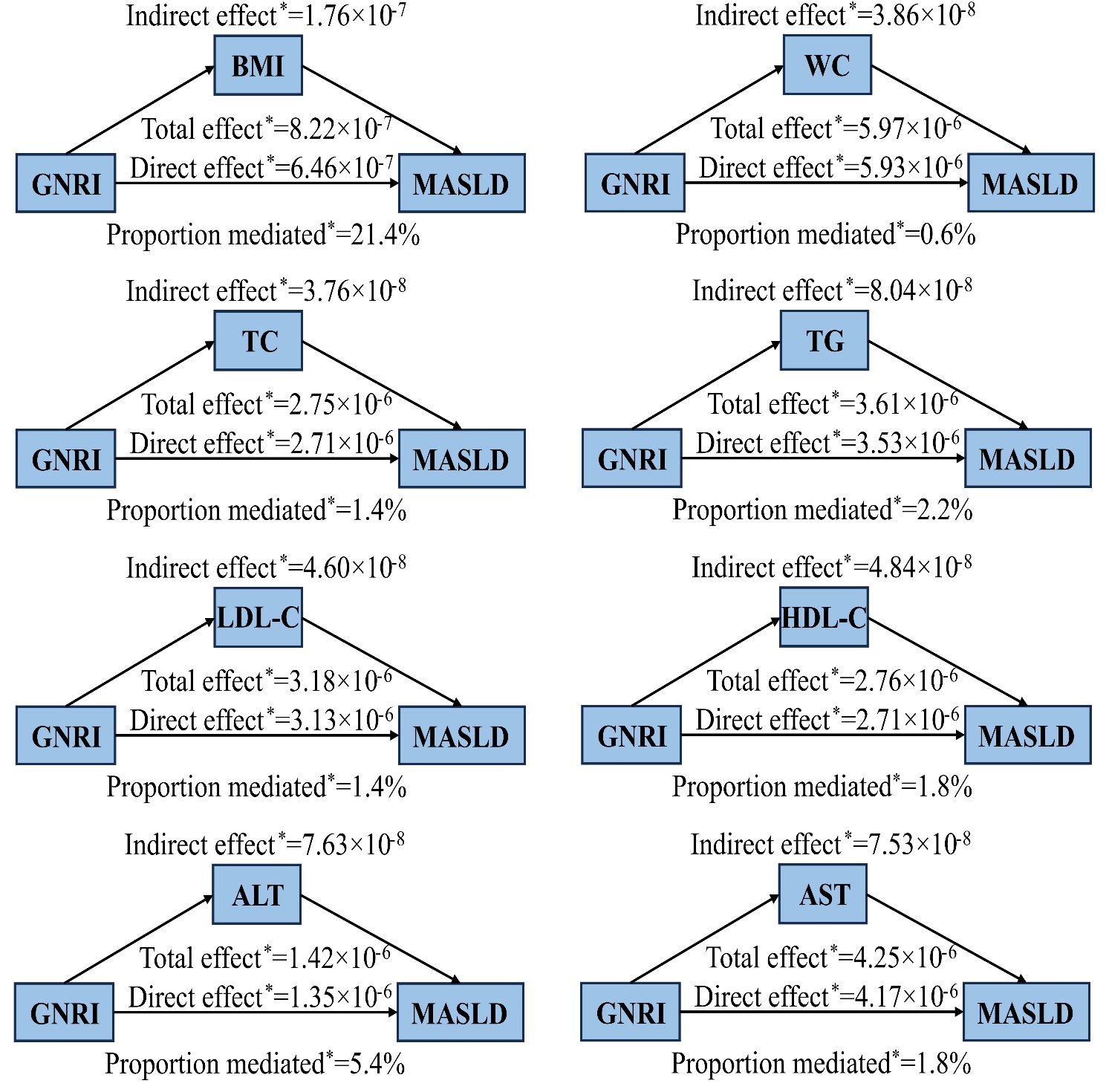
****Supplementary Figure 3. Mediation analysis for the associations between GNRI and MASLD (2023 International Multi-Society Consensus on MASLD) in the elderly population.**

* *P* < 0.05.

Adjusted for gender, age group, marital status, educational attainment, smoking status, drinking status, BMI group, WC, ALT, AST, UA, TC, TG, HDL-C, hypertension and glycemic status. In the process of mediation analysis, the mediator variables are excluded from the adjustment to accurately estimate the direct and indirect effects between the GNRI and MASLD. Abbreviations: ALT, alanine aminotransferase; AST, aspartate aminotransferase; BMI, body mass index; GNRI, geriatric nutritional risk index; HDL-C, high-density lipoprotein cholesterol; LDL-C, low-density lipoprotein cholesterol; MASLD, metabolic dysfunction-associated steatotic liver disease; TC, total cholesterol; TG, triglycerides; WC, waist circumference; UA, uric acid.

## Supplementary Tables

Supplementary Table 1. Characteristics among individuals with MASLD or non-MASLD (2023 International Multi-Society Consensus on MASLD)

| **Characteristics** | **Total (n=7,628)** | **MASLD (n=2,863)** | **Non-MASLD (n=4,765)** | ***P* value** |
| --- | --- | --- | --- | --- |
| Age (year) | 67.00 (64.00, 71.00) | 67.00 (63.00, 71.00) | 67.00 (64.00, 72.00) | <0.001 |
| Age group (year) |  |  |  |  |
| < 75 | 6593 (86.4%) | 2566 (89.6%) | 4027 (84.5%) | <0.001 |
| ≥ 75 | 1035 (13.6%) | 297 (10.4%) | 738 (15.5%) |  |
| Gender |  |  |  |  |
| Male | 3080 (40.4%) | 924 (32.3%) | 2156 (45.3%) | <0.001 |
| Female | 4548 (59.6%) | 1939 (67.7%) | 2609 (54.7%) |  |
| Ethnic group |  |  |  |  |
| Han ethnicity | 7610 (99.8%) | 2853 (99.7%) | 4757 (99.8%) | 0.114 |
| Ethnic minorities | 18 (0.2%) | 10 (0.3%) | 8 (0.2%) |  |
| Educational attainment |  |  |  |  |
| Primary school and below | 3954 (51.8%) | 1541 (53.8%) | 2413 (50.6%) | 0.057 |
| Junior high school | 2105 (27.6%) | 764 (26.7%) | 1341 (28.1%) |  |
| Senior high school | 1218 (16.0%) | 435 (15.2%) | 783 (16.4%) |  |
| Undergraduate and above | 351 (4.6%) | 123 (4.3%) | 228 (4.8%) |  |
| Marital status |  |  |  |  |
| Unmarried | 158 (2.1%) | 85 (3.0%) | 73 (1.5%) | <0.001 |
| Married or cohabiting | 6353 (83.3%) | 2363 (82.5%) | 3990 (83.7%) |  |
| Divorced or separated | 130 (1.7%) | 63 (2.2%) | 67 (1.4%) |  |
| Widowed | 987 (12.9%) | 352 (12.3%) | 635 (13.3%) |  |
| Smoking status |  |  |  |  |
| Never smokers | 6187 (81.1%) | 2478 (86.6%) | 3709 (77.8%) | <0.001 |
| Current smokers | 827 (10.8%) | 208 (7.3%) | 619 (13.0%) |  |
| Former smokers | 614 (8.1%) | 177 (6.2%) | 437 (9.2%) |  |
| Drinking status |  |  |  |  |
| Non-drinkers | 6961 (91.3%) | 2710 (94.7%) | 4251 (89.2%) | <0.001 |
| Regular drinkers | 667 (8.7%) | 153 (5.3%) | 514 (10.8%) |  |
| BMI (kg/m^2^) | 23.82 (21.85, 25.99) | 25.15 (23.24, 27.30) | 23.06 (21.10, 25.08) | <0.001 |
| BMI group (kg/m^2^) |  |  |  |  |
| <24.0 | 4001 (52.5%) | 1008 (35.2%) | 2993 (62.8%) | <0.001 |
| ≥24.0 | 3627 (47.6%) | 1855 (64.8%) | 1772 (37.2%) |  |
| WC (cm) | 86.30 (80.20, 92.20) | 89.60 (84.00, 95.60) | 84.30 (78.20, 90.00) | <0.001 |
| FBG (mmol/L) | 5.36 (4.92, 6.00) | 5.60 (5.10, 6.53) | 5.23 (4.86, 5.76) | <0.001 |
| ALT (U/L) | 18.30 (14.20, 25.00) | 21.40 (16.30, 30.20) | 16.90 (13.40, 22.20) | <0.001 |
| AST (U/L) | 22.20 (19.30, 26.30) | 23.10 (19.80, 27.80) | 21.80 (19.10, 25.40) | <0.001 |

(continued)

Supplementary Table 1. Characteristics among individuals with MASLD or non-MASLD (2023 International Multi-Society Consensus on MASLD) (continued)

| **Characteristics** | **Total (n=7,628)** | **MASLD (n=2,863)** | **Non-MASLD (n=4,765)** | ***P* value** |
| --- | --- | --- | --- | --- |
| TC (mmol/L) | 5.37 (4.67, 6.12) | 5.50 (4.76, 6.27) | 5.30 (4.62, 6.04) | <0.001 |
| TG (mmol/L) | 1.46 (1.06, 2.08) | 1.78 (1.31, 2.47) | 1.30 (0.96, 1.80) | <0.001 |
| LDL-C (mmol/L) | 3.06 (2.51, 3.61) | 3.17 (2.62, 3.75) | 3.00 (2.46, 3.53) | <0.001 |
| HDL-C (mmol/L) | 1.28 (1.09, 1.50) | 1.21 (1.05, 1.40) | 1.33 (1.13, 1.56) | <0.001 |
| UA (µmol/L) | 347.90 (288.90, 417.00) | 366.00 (308.00, 437.00) | 333.70 (279.80, 402.00) | <0.001 |
| Hypertension |  |  |  |  |
| No | 2948 (38.7%) | 962 (33.6%) | 1986 (41.7%) | <0.001 |
| Yes | 4680 (61.4%) | 1901 (66.4%) | 2779 (58.3%) |  |
| Glycemic status |  |  |  |  |
| Normal | 3956 (51.9%) | 1003 (35.0%) | 2953 (62.0%) | <0.001 |
| Pre-diabetes | 1959 (25.7%) | 947 (33.1%) | 1012 (21.2%) |  |
| Diabetes | 1713 (22.5%) | 913 (31.9%) | 800 (16.8%) |  |
| GNRI | 107.51 (104.79, 109.77) | 108.71 (106.62, 110.79) | 106.62 (103.73, 109.00) | <0.001 |
| GNRI grades of nutrition-related risk |  |  |  |  |
| Major risk | 4 (0.1%) | 0 (0.0%) | 4 (0.1%) | <0.001 |
| Moderate risk | 26 (0.3%) | 0 (0.0%) | 26 (0.6%) |  |
| Low risk | 229 (3.0%) | 11 (0.4%) | 218 (4.6%) |  |
| No risk | 7369 (96.6%) | 2852 (99.6%) | 4517 (94.8%) |  |
| GNRI quartiles group |  |  |  |  |
| Q1 | 1907 (25.0%) | 339 (11.8%) | 1568 (32.9%) | <0.001 |
| Q2 | 1857 (24.3%) | 642 (22.4%) | 1215 (25.5%) |  |
| Q3 | 1956 (25.6%) | 833 (29.1%) | 1123 (23.6%) |  |
| Q4 | 1908 (25.0%) | 1049 (36.6%) | 859 (18.0%) |  |

Data are presented as median (P_25_, P_75_) for continuous variables and frequency (percentages) for categorical variables.

Abbreviations: ALT, alanine aminotransferase; AST, aspartate aminotransferase; BMI, body mass index; GNRI, geriatric nutritional risk index; HDL-C, high-density lipoprotein cholesterol; LDL-C, low-density lipoprotein cholesterol; MASLD, metabolic dysfunction-associated steatotic liver disease; TC, total cholesterol; TG, triglycerides; OR, odds ratio; Q, quartile; WC, waist circumference; UA, uric acid.

**Supplementary Table 2. Association between the GNRI and MASLD (2023 International Multi-Society Consensus on MASLD) based on binary logistic regression analysis**

| **Model** | **GNRI Continuous**  **OR (95% CI)** | **GNRI Quartiles OR (95% CI)** | | | | ***P* for trend** |
| --- | --- | --- | --- | --- | --- | --- |
|  |  | **Q1** | **Q2** | **Q3** | **Q4** |  |
| Crude ^a^ | 1.19  (1.17, 1.20) | 1.00 | 2.44  (2.10, 2.84) | 3.43  (2.96, 3.98) | 5.65  (4.87, 6.55) | <0.001 |
| Model 1 ^b^ | 1.18  (1.17, 1.20) | 1.00 | 2.33  (2.00, 2.72) | 3.24  (2.79, 3.76) | 5.46  (4.70, 6.35) | <0.001 |
| Model 2 ^c^ | 1.14  (1.13, 1.16) | 1.00 | 1.76  (1.50, 2.06) | 2.22  (1.89, 2.59) | 3.66  (3.12, 4.29) | <0.001 |
| Model 3 ^d^ | 1.11  (1.09, 1.13) | 1.00 | 1.58  (1.33, 1.87) | 1.92  (1.62, 2.27) | 2.75  (2.32, 3.26) | <0.001 |

^a^ Non-adjusted.

^b^ Adjusted for gender, age group, ethnic group, marital status and educational attainment.

^c^ Further adjusted for smoking status, drinking status, BMI group and WC.

^d^ Further adjusted for ALT, AST, UA, TC, TG, HDL-C, hypertension and glycemic status.

Abbreviations: ALT, alanine aminotransferase; AST, aspartate aminotransferase; BMI, body mass index; CI, confidence interval; GNRI, geriatric nutritional risk index; HDL-C, high-density lipoprotein cholesterol; MASLD, metabolic dysfunction-associated steatotic liver disease; TC, total cholesterol; TG, triglycerides; OR, odds ratio; Q, quartile; WC, waist circumference; UA, uric acid.

**Supplementary Table 3. Stratified analysis of the association between the GNRI and MASLD (2023 International Multi-Society Consensus on MASLD)**

| **Characteristics** | **GNRI Continuous**  **OR (95% CI)** | **GNRI Quartiles OR (95% CI)** | | | | ***P* for trend** |
| --- | --- | --- | --- | --- | --- | --- |
|  |  | **Q1** | **Q2** | **Q3** | **Q4** |  |
| Gender |  |  |  |  |  |  |
| Male | 1.13  (1.10, 1.16) | 1.00 | 1.81  (1.36, 2.40) | 2.39  (1.80, 3.17) | 3.38  (2.56, 4.45) | < 0.001 |
| Female | 1.10  (1.07, 1.12) | 1.00 | 1.46  (1.18, 1.81) | 1.69  (1.37, 2.09) | 2.33  (1.87, 2.89) | < 0.001 |
| Age (year) |  |  |  |  |  |  |
| <75 | 1.11  (1.09, 1.13) | 1.00 | 1.55  (1.29, 1.87) | 1.88  (1.57, 2.26) | 2.72  (2.26, 3.26) | < 0.001 |
| ≥75 | 1.12  (1.07, 1.17) | 1.00 | 1.64  (1.05, 2.56) | 2.07  (1.34, 3.21) | 3.00  (1.85, 4.86) | < 0.001 |
| BMI (kg/m^2^) |  |  |  |  |  |  |
| <24.0 | 1.14  (1.11, 1.16) | 1.00 | 2.26  (1.75, 2.91) | 2.56  (1.98, 3.31) | 3.96  (3.06, 5.14) | < 0.001 |
| ≥24.0 | 1.09  (1.06, 1.11) | 1.00 | 1.12  (0.88, 1.43) | 1.40  (1.11, 1.77) | 1.93  (1.52, 2.45) | < 0.001 |
| Smoking status |  |  |  |  |  |  |
| Never smokers | 1.11  (1.09, 1.13) | 1.00 | 1.60  (1.33, 1.93) | 1.93  (1.60, 2.32) | 2.63  (2.18, 3.18) | < 0.001 |
| Current smokers | 1.14  (1.09, 1.20) | 1.00 | 1.85  (1.06, 3.23) | 2.19  (1.22, 3.95) | 3.96  (2.31, 6.79) | < 0.001 |
| Former smokers | 1.12  (1.05, 1.19) | 1.00 | 1.17  (0.59, 2.32) | 1.67  (0.89, 3.14) | 2.65  (1.41, 4.99) | 0.001 |
| Drinking status |  |  |  |  |  |  |
| Non-drinkers | 1.11  (1.09, 1.13) | 1.00 | 1.54  (1.29, 1.84) | 1.82  (1.53, 2.17) | 2.64  (2.21, 3.15) | < 0.001 |
| Regular drinkers | 1.11  (1.05, 1.18) | 1.00 | 2.20  (1.07, 4.54) | 3.39  (1.69, 6.78) | 4.01  (2.00, 8.04) | < 0.001 |
| Hypertension |  |  |  |  |  |  |
| No | 1.10  (1.08, 1.13) | 1.00 | 1.66  (1.26, 2.19) | 1.81  (1.37, 2.39) | 2.50  (1.88, 3.31) | < 0.001 |
| Yes | 1.12  (1.09, 1.14) | 1.00 | 1.52  (1.22, 1.89) | 1.95  (1.57, 2.41) | 2.89  (2.34, 3.58) | < 0.001 |
| Glycemic status |  |  |  |  |  |  |
| Normal | 1.15  (1.12, 1.18) | 1.00 | 1.66  (1.29, 2.14) | 1.97  (1.53, 2.53) | 3.47  (2.68, 4.50) | < 0.001 |
| Pre-diabetes | 1.09  (1.06, 1.12) | 1.00 | 1.57  (1.12, 2.22) | 1.79  (1.29, 2.50) | 2.31  (1.67, 3.20) | < 0.001 |
| Diabetes | 1.08  (1.05, 1.12) | 1.00 | 1.42  (1.02, 1.97) | 1.97  (1.41, 2.73) | 2.26  (1.64, 3.10) | < 0.001 |

Adjusted for gender, age group, marital status, educational attainment, smoking status, drinking status, BMI group, WC, ALT, AST, UA, TC, TG, HDL-C, hypertension and glycemic status. The strata variable was not included when stratifying by itself.

Abbreviations: ALT, alanine aminotransferase; AST, aspartate aminotransferase; BMI, body mass index; CI, confidence interval; GNRI, geriatric nutritional risk index; HDL-C, high-density lipoprotein cholesterol; MASLD, metabolic dysfunction-associated steatotic liver disease; TC, total cholesterol; TG, triglycerides; OR, odds ratio; Q, quartile; WC, waist circumference; UA, uric acid.

**Supplementary Table 4. Multiple logistic regression analysis of the association between GNRI and MASLD subtypes using non-MASLD as the reference group (2023 International Multi-Society Consensus on MASLD)**

| **MASLD subtypes** | **GNRI Continuous**  **OR (95% CI)** | **GNRI Quartiles OR (95% CI)** | | | | ***P* for trend** |
| --- | --- | --- | --- | --- | --- | --- |
|  |  | **Q1** | **Q2** | **Q3** | **Q4** |  |
| MASLD (diabetes) (n=913) |  |  |  |  |  |  |
| Crude ^a^ | 1.18  (1.16, 1.20) | 1.00 | 2.02  (1.59, 2.56) | 2.91  (2.31, 3.65) | 5.08  (4.06, 6.34) | < 0.001 |
| Model 1 ^b^ | 1.18  (1.16, 1.20) | 1.00 | 1.96  (1.54, 2.49) | 2.82  (2.24, 3.55) | 5.02  (4.01, 6.29) | < 0.001 |
| Model 2 ^c^ | 1.18  (1.16, 1.20) | 1.00 | 1.95  (1.53, 2.48) | 2.81  (2.23, 3.53) | 4.99  (3.99, 6.26) | < 0.001 |
| Model 3 ^d^ | 1.14  (1.11, 1.16) | 1.00 | 1.52  (1.18, 1.96) | 2.04  (1.60, 2.60) | 3.37  (2.66, 4.28) | < 0.001 |
| MASLD (overweight/obesity) (n=1,520) |  |  |  |  |  |  |
| Crude ^a^ | 1.21  (1.19, 1.23) | 1.00 | 2.97  (2.41, 3.66) | 4.41  (3.60, 5.39) | 7.34  (6.01, 8.96) | < 0.001 |
| Model 1 ^b^ | 1.21  (1.19, 1.23) | 1.00 | 2.82  (2.29, 3.48) | 4.13  (3.37, 5.06) | 7.03  (5.75, 8.61) | < 0.001 |
| Model 2 ^c^ | 1.21  (1.18, 1.23) | 1.00 | 2.79  (2.26, 3.44) | 4.06  (3.31, 4.98) | 6.93  (5.66, 8.49) | < 0.001 |
| Model 3 ^d^ | 1.17  (1.15, 1.19) | 1.00 | 2.29  (1.85, 2.85) | 3.14  (2.55, 3.88) | 5.06  (4.10, 6.24) | < 0.001 |
| MASLD  (lean metabolic disorder)  (n=430) |  |  |  |  |  |  |
| Crude ^a^ | 1.15  (1.13, 1.18) | 1.00 | 2.11  (1.56, 2.86) | 2.34  (1.73, 3.17) | 3.19  (2.35, 4.32) | < 0.001 |
| Model 1 ^b^ | 1.15  (1.12, 1.17) | 1.00 | 1.97  (1.45, 2.69) | 2.16  (1.59, 2.93) | 3.00  (2.21, 4.08) | < 0.001 |
| Model 2 ^c^ | 1.15  (1.12, 1.17) | 1.00 | 1.98  (1.45, 2.69) | 2.18  (1.60, 2.96) | 3.02  (2.22, 4.10) | < 0.001 |
| Model 3 ^d^ | 1.15  (1.12, 1.17) | 1.00 | 1.69  (1.24, 2.31) | 1.80  (1.32, 2.46) | 2.42  (1.76, 3.31) | < 0.001 |

^a^ Non-adjusted.

^b^ Adjusted for gender, age group, marital status, ethnic group and educational attainment.

^c^ Further adjusted for smoking status and drinking status.

^d^ Further adjusted for ALT, AST, UA, TC, TG, HDL-C and hypertension.

Note: Results are from multiple logistic regression analyses with non-MASLD as the reference group.

Abbreviations: ALT, alanine aminotransferase; AST, aspartate aminotransferase; CI, confidence interval; GNRI, geriatric nutritional risk index; HDL-C, high-density lipoprotein cholesterol; MASLD, metabolic dysfunction-associated steatotic liver disease; TC, total cholesterol; TG, triglycerides; OR, odds ratio; Q, quartile; UA, uric acid.
